# Effectiveness of Bromelain in the control of postoperative pain after periodontal surgery: A crossover randomized clinical trial

**DOI:** 10.34172/japid.2023.002

**Published:** 2023-05-07

**Authors:** Masoumeh Faramarzi, Mehrnoosh Sadighi, Adileh Shirmohamadi, Reza Kazemi, Mahsa Zohdi

**Affiliations:** ^1^Department of Periodontics, Dental and Periodontal Research Center, Tabriz University of Medical Sciences, Tabriz, Iran; ^2^School of Dentistry, Tabriz University of Medical Sciences, Tabriz, Iran; ^3^Students Research Committee, Tabriz University of Medical Science, Tabriz, Iran

**Keywords:** Bromelain, Oral surgery, Periodontics, Visual analog scale

## Abstract

**Background.:**

This study aimed to compare the analgesic effects of Ibuprofen and bromelain after periodontal surgery.

**Methods.:**

A double-blinded crossover clinical trial was conducted on 22 patients needing two crown lengthening surgeries without bone surgery or with limited bone surgery on two quadrants of the maxilla, with control and test sides. Each quadrant was randomly assigned to bromelain (500 GUD) or ibuprofen (400 mg). Immediately after the surgery and 6 hours after it, the first dose of the drugs was packaged in the same capsules in A and B. Postoperative pain was evaluated during the first 8 hours and on the following day using a visual analog scale (VAS).

**Results.:**

Using the VAS, the Ibuprofen group showed significantly lower mean pain scores than the bromelain group at 4 hours (*P*=0.047). In contrast, there were no significant differences between the two groups at 2, 6, 8, 10, 12, 24, and 48 hours (*P*>0.05).

**Conclusion.:**

The effectiveness of bromelain for pain control following periodontal surgery was comparable to that of Ibuprofen. Therefore, bromelain can be an efficient replacement for ibuprofen in managing pain after periodontal surgery, especially in patients with gastric ulceration and increased bleeding tendency.

## Introduction

 Discomfort, edema, and pain following periodontal surgery are common findings, especially during the first 24 hours after the surgical procedure. This pain can be an example of acute dental pain with mild to moderate severity.^1,2^ The severity of the pain depends on various factors such as the type of periodontal surgery (mucogingival surgery, periodontal flap, and bone surgery), the amount of trauma to the tissues, the type of anesthetic drug, psychological conditions, and the patient’s stress.^3,4^

 Many inflammatory mediators are released after damage to periodontal tissues.^1^ Two types of cyclooxygenase (COX-1 and COX-2) have been identified.^2,4^ COX-1, a structural component in many tissues of the body, results in homeostasis in the body by producing prostaglandins. Its main functions include protecting the gastrointestinal tract, increasing renal function, affecting the central nervous system, and increasing the circulatory system’s permeation.^5,6^ COX-2, an inducible form, produces prostaglandins that mediate the inflammatory response and pain-signaling transmissions. It is initially responsible for producing prostaglandins which accelerate vasodilation, increase vessel permeability, and decrease pain threshold.^2-7^ Conventional non-selective nonsteroidal anti-inflammatory (NSAIDs) drugs such as ibuprofen inhibit COX-1 and COX-2.^5^ Although this drug significantly improves inflammation and pain, its long-term use has side effects, such as gastric and renal toxicity. For this reason, selective inhibitors of COX-2 have been developed to reduce these side effects.^5,6^ Bromelain is a water-based product derived from the stem and fruit of pineapple with plenty of proteolytic enzymes, whose composition varies depending on the source and purification method. Bromelain directly affects mediators of pain such as bradykinin. Also, the analgesic and anti-inflammatory properties are closely interrelated. It has been shown that this fibrinolytic element increases the absorption of edema by blood circulation. This element reduces swelling, bruising, pain and duration of postoperative healing after trauma or surgery. Evidence has shown that bromelain removes edema by fibrin degradation. In addition, bromelain inhibits the synthesis of pro-inflammatory prostaglandins, especially prostaglandin E_2_.^8-10^ Bromelain is used instead of NSAIDs in patients with osteoarthritis.^11^

 Majid and Al-Mashhadani^12^ evaluated the effect of oral bromelain versus oral diclofenac sodium with placebo on pain, quality of life, swelling, and trismus after the surgical removal of impacted lower third molars. The individuals who had used diclofenac and bromelain had considerably lower mean pain scores than those using a placebo. Bromelain’s effect on postoperative pain was comparable to that of diclofenac sodium. Zatuchni and Colombi^13^ evaluated the effect of bromelain on the pain intensity of episiotomy and showed that the severity of pain, inflammation, and edema in individuals treated by bromelain was significantly lower than in the group using a placebo, and wound healing was faster in these individuals. Hotz et al^14^ evaluated the efficacy of bromelain in reducing postoperative pain and swelling. They reported an important anti-inflammatory and anti-edematous effect of bromelain. According to statistical analyses, the inflammatory responses in the group treated with bromelain were significantly less than the control group.

 In another study by Inchingolo et al^15^ to assess the efficacy of bromelain in controlling edema and its related pain in the inflamed area after upper third molar exodontia, the effectiveness of bromelain in treating postoperative edema after third molar surgery was reported.

 The present study investigated the effect of bromelain on pain relief after periodontal surgery using a prospective, double-blind clinical trial to select a suitable drug for pain relief in these patients.

## Methods

###  Study population 

 This study received approval from the Human Research Ethics Committee of Tabriz University of Medical Sciences under the code IR.TBZMED.REC.1395.496. This randomized, double-blind crossover clinical trial was conducted on 22 patients (12 men and 10 women) aged 35‒50 years from those referring to the Department of Periodontics, Faculty of Dentistry, Tabriz University of Medical Sciences. All the patients were selected according to inclusion and exclusion criteria. The entire study process was explained to the patients. All the patients agreed to participate by signing a written informed consent form that thoroughly explained the steps of the study. The clinical trial registration number of this research was IRCT201608301760N46.

###  Study design

**Figure 1:**
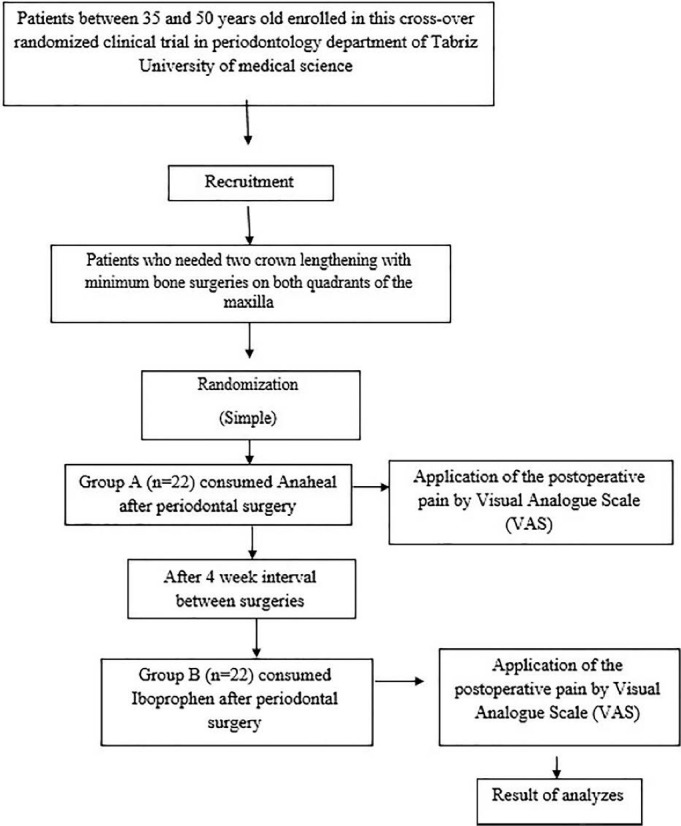
Flow chart of the study protocol

 This study was performed on patients needing two crown lengthening surgeries on both quadrants of the maxilla. To match the two surgical areas as much as possible, patients without bone surgery or with minimum bone surgery were included in the study, and the surgeries were performed by one surgeon. The investigation was designed, analyzed, and interpreted according to the Consolidated Standards of Reporting Trials (CONSORT) (Figure 1).

 The exclusion criteria were as follows: any sensitivity to these two drugs or other nonsteroidal anti-inflammation drugs, asthmatic patients, heart disease history, pregnancy, patients with indigestion systemic diseases like peptic ulcers, renal or hepatic diseases, coagulative disorders, patients with severe anxiety during dental visits, patients taking analgesics in the previous 48 hours, patients > 50 years of age, individuals using alcohol and addictive drugs.

 Since anxiety varies from one patient to another and may affect their pain severity, patients were asked to complete Modified Dental Anxiety Scale (MDAS) questionnaire. This questionnaire includes five questions and five answers for each question. Each item has five answers, and the range of answers is from “not anxious” to “extremely anxious.” The responses are scored from 1 to 5 in ascending order in terms of increasing intensity of dental anxiety. By rating all five items, the total score of all questions for the scale will vary from 5 to 25. The cutoff score of ≥ 19 indicates patients with high dental anxiety or possibly dental phobia.^16^

 Ibuprofen (400 mg) (Hakim Pharmaceuticals Co., Tehran, Iran) and Anaheal GDU 500 (Tasnim Pharmaceutical Co., Tehran, Iran) were prepared as the drugs needed for this study. Each capsule of Anaheal contains 200 mg (500 GDU) bromelain (2500 GDU/g). The two medications were poured into similar capsules and coded into groups A and B by a pharmacologist (PZ). For each patient, two surgeries were conducted in two different jaw quadrants with a 4-week interval. Patients underwent crown lengthening by one resident of periodontics (AM) with sufficient experience.

 Only patients who needed crown lengthening of maxillary teeth were selected to unify the study groups and eliminate possible confounding factors during surgery in various parts of the oral cavity. The duration of surgery in this study was 45‒60 minutes; otherwise, the patients would be excluded. All the surgeries were performed in the morning. The surgeon was allowed to use two capsules of 2% lidocaine with 1:100 000 epinephrine for each patient. The patients consumed group A drug 1 hour before the first surgery and then every 6 hours. Likewise, they used group B drug for the second surgery. The patients’ pain intensity was evaluated 2, 4, 6, 8, 10, 12, 24, and 48 hours after the surgery using visual analog scale (VAS).^17^ The VAS was presented to the patients as a 10-cm ruler, with the point at the left end of the VAS range representing complete analgesia and the point at the right end representing the highest pain imaginable. Patients expressed their pain severity by marking a point somewhere between the two end points at 2, 4, 6, 8, 10, 12, 24, and 48 hours postoperatively, which was recorded in the relevant forms. For ethical purposes, acetaminophen codeine was prescribed for all the patients as a supplementary analgesic. The patients were justified to use it only if they had intolerable pain.

###  Statistical analysis

 The data were analyzed using SPSS 17.0 using descriptive statistics (means and standard deviations). The differences between the two groups were analyzed using the Mann-Whitney U test for independent samples at a significance level of *P* < 0.05.

## Results

 The total number of patients was 22 (12 men and 10 women) with 44 quadrants, with a mean age of 45.82 ± 2.8 years. All the participants completed the study, and no side effects were reported for each drug. In addition, the amount of local anesthesia used, the type and extent of surgery, and the duration of surgery were the same for all the subjects.

**Figure 2:**
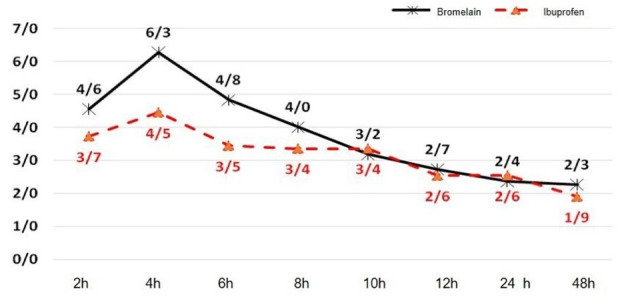
Comparison of the VAS scores between the Bromelain and Ibuprofen groups after surgery

Table 1 shows the means ± SD for comparing pain scores between the two study groups using the VAS. At 2-, 4-, 6-, 8-, 10-, 12-, 24-, and 48-hour postoperative intervals, no significant differences were observed in pain between the two groups. However, at 4 hours, the ibuprofen group showed a signiﬁcantly lower mean pain score compared to the bromelain group (Figure 2) (*P* < 0.05).

**Table 1:** Frequencies of scores given by patients

**Time**	**Test group**	**Control group**	***P*** ** value**
2 hours	4.55 ± 2.16	3.73 ± 1.79	0.394
4 hours	6.27 ± 2.24	4.45 ± 2.38	0.047*
6 hours	4.82 ± 1.83	3.45 ± 1.81	0.112
8 hours	4.00 ± 2.00	3.36 ± 1.91	0.452
10 hours	3.18 ± 1.54	3.36 ± 2.58	0.673
12 hours	2.73 ± 1.19	2.55 ± 1.51	0.503
24 hours	2.36 ± 0.92	2.55 ± 1.69	0.746
48 hours	2.27 ± 0.90	1.91 ± 0.36	0.281

## Discussion

 Postoperative pain is unpleasant. Trauma and tissue damage during surgery lead to the destruction of cell membranes and the release of phospholipids that turn into prostaglandins, thromboxane, and other metabolites through COX-1 and COX-2 enzymes, ultimately causing pain.^18^ Many drugs with different mechanisms have been introduced to prevent or reduce pain. One of the most common drugs for pain control is NSAIDs which reduce pain by inhibiting prostaglandins.^19^ Recently, a new analgesic agent, referred to as bromelain, has been marketed in capsule form. It is extracted from the root of pineapple fruit. Several studies have supported the usefulness of oral administration of bromelain in controlling pain and swelling after surgery.^8,20^ It has been shown that bromelain has analgesic and anti-inflammatory properties in addition to its antithrombotic, anti-edematous, and fibrinolytic effects. Experimental evidence shows that bromelain’s anti-inflammatory function is mediated through several factors: by increasing serum fibrinolytic activity, reducing plasma fibrinogen levels, and decreasing bradykinin levels, which lead to reduced edema and pain.^21^ It is believed that the analgesic properties of bromelain are due to its direct effect on pain mediators such as bradykinin and its indirect anti-inflammatory function, including a reduction in edema, debris, and immune complexes, which leads to pain relief.^22,23^

 The present randomized, double-blind crossover study was designed to compare the analgesic effects of bromelain with a commonly used analgesic agent, ibuprofen 400 mg, to evaluate the possibility of using this drug as an alternative to ibuprofen to decrease its side effects. The intensity of the pain depends on several factors, including the type of dental treatment, such as endodontic treatment or periodontal surgery, the type of periodontal surgery, the duration of surgery, the psychological condition of the patient, the patient’s gender, and the extent of tissue trauma during surgery.^24^ Therefore, in the present study, we tried to match the subjects enrolled regarding age, gender, anxiety, type, and duration of surgery. Since anxiety varies from one patient to another, the patients were asked to complete the MDAS questionnaire.^25^ Subjects whose total scores were > 19 were excluded.

 In the present study, the severity of pain 4, 6, 8, 10, 12, 24, and 48 hours after the surgery (based on VAS) was not significantly different between the bromelain and ibuprofen groups. The main finding was that bromelain’s analgesic effects were similar to ibuprofen, which could justify using bromelain as an alternative to NSAIDs. These findings are consistent with other studies in this respect. Majid and Al-Mashhadani^12^ evaluated the effects of bromelain and Diclofenac sodium on pain relief after the surgical removal of third molar teeth. Both groups showed significant pain relief compared to the placebo group.

 On the other hand, Inchingolo et al^15^ compared bromelain (40 mg every 6 hours for 6 days) with ketoprofen (100 mg every 12 hours for 6 days) and found no differences between them, concluding that bromelain is as effective as NSAIDs in relieving postoperative inflammation. In addition, de la Barrera-Núñez et al^26^ evaluated the effect of bromelain administered orally in the postoperative pain control after removing impacted lower molars. All the patients in this study received the rescue medications. However, the test group was administered three tablets of bromelain per day during the first three days and two tablets per day from the fourth to the seventh day. The control group was given a placebo. There were no statistically significant differences between the treatment groups. The differences between studies might be primarily due to a lack of knowledge of the effective dose of bromelain indicated for postoperative pain control. It has been demonstrated that the effect of bromelain is dose-dependent. The benefits of bromelain have been demonstrated at a small dose of 160 mg, but in most conditions, the best results are achieved at doses of 750‒1000 mg/d in four divided doses.^27^ However, the results of the present study showed that pain intensity four hours after surgery in the bromelain group was significantly more than in the ibuprofen group. This finding can be attributed to the pharmacokinetic differences between the two drugs. Ibuprofen acts centrally and peripherally, inhibiting cyclooxygenase in the brain and peripheral tissues. Its maximum effect is achieved at a dose of 400 mg. An oral dose of 400 mg is rapidly absorbed and reaches a peak plasma level in 30‒60 minutes. Its half-life in the plasma is 1.6‒2.5 hours.^28^ According to the plasma concentration curve, there is an average of 10.8 µg of bromelain for 3‒51 hours. The estimated plasma half-life was 6‒9 hours.^29^ This pharmacokinetic difference between the two drugs can justify the severity of pain two hours after surgery for bromelain versus ibuprofen. When ibuprofen is taken postoperatively, the plasma concentration is expected to be below optimal levels 5‒6 hours after dosing.^30^

 In this study, pain intensity was recorded for the first 8 hours and on the day after surgery. An 8-hour period seems appropriate for pain intensity assessment because it properly covers the duration of action of both drugs. There is also evidence that pain levels after periodontal surgery are the greatest within the immediate 11-hour postoperative period.^31^

 Our study was limited by a small number of participants. Nevertheless, this study highlights the need for further large-scale and long-term clinical trials to examine the efficacy of bromelain in pain relief. In addition, it is suggested that further studies be carried out to evaluate the side effects of these two medications after periodontal surgeries.

## Conclusion

 Bromelain is an efficient alternative to ibuprofen to manage pain after periodontal surgery, especially in patients with gastric ulceration and increased bleeding. Additional studies with a larger sample size and different doses of bromelain are recommended to determine a better medication.

## Acknowledgments

 None.

## Availability of Data

 The raw/processed data required to reproduce these findings can be shared after publication by requesting from the corresponding author.

## Competing Interests

 Adileh Shirmohammadi is the editor-in-chief of JAPID at the time of publication. The authors declare that they have no other competing interests with regards to authorship and/or publication of this work.

## Ethical Approval

 This study received approval from the Human Research Ethics Committee of Tabriz-Iran University of Medical Science under ethics number IR.TBZMED.REC.1395.496.

## Funding

 Not applicable.
